# Dressing Tool Condition Monitoring through Impedance-Based Sensors: Part 2—Neural Networks and K-Nearest Neighbor Classifier Approach

**DOI:** 10.3390/s18124453

**Published:** 2018-12-16

**Authors:** Pedro Junior, Doriana M. D’Addona, Paulo Aguiar, Roberto Teti

**Affiliations:** 1 Faculdade de Engenharia, UNESP-University Estadual Paulista, Bauru, Departamento de Engenharia Elétrica, Av. Eng. Luiz Edmundo C. Coube 14-01, 17033-360 Bauru–SP, Brazil; pedrojunior5@aedu.com (P.J.); aguiarpr@feb.unesp.br (P.A.); 2 Dipartimento di Ingegneria Chimica, Università degli Studi di Napoli Federico II, dei Materiali e della Produzione Industriale; 80138 Napoli NA, Italy; roberto.teti@unina.it

**Keywords:** sensor monitoring, tool condition monitoring, piezoelectric sensors, electromechanical impedance, dressing, grinding process, neural networks, MLNN, *k*-NN

## Abstract

This paper presents an approach for impedance-based sensor monitoring of dressing tool condition in grinding by using the electromechanical impedance (EMI) technique. This method was introduced in Part 1 of this work and the purpose of this paper (Part 2) is to achieve an optimal selection of the excitation frequency band based on multi-layer neural networks (MLNN) and k-nearest neighbor classifier (*k*-NN). The proposed approach was validated on the basis of dressing tool condition information obtained from the monitoring of experimental dressing tests with two industrial stationary single-point dressing tools. Moreover, representative damage indices for diverse damage cases, obtained from impedance signatures at different frequency bands, were taken into account for MLNN data processing. The intelligent system was able to select the most damage-sensitive features based on optimal frequency band. The best models showed a general overall error lower than 2%, thus robustly contributing to the efficient automation of grinding and dressing operations. The promising results of this study foster the EMI-based sensor monitoring approach to fault diagnosis in dressing operations and its effective implementation for industrial grinding process automation.

## 1. Introduction

Grinding is a finishing process for complex geometry machined parts which requires high precision material removal with minimum number of defects to ensure high accuracy and quality in the final workpiece [1]. However, the success of any grinding process is critically dependent on the performance of the tool (grinding wheel). The latter is decisively conditioned by the dressing of its surface necessary to remodel the wheel when there is excessive deviation from its original shape due to wear [2]. The main objective of the dressing operation is to generate a satisfactory grinding wheel topography, which has significant impact on the grinding force, energy, temperatures, wheel wear and part surface finish. Moreover, dressing errors can adversely affect the quality of the grinding wheel as the result of tool wear and other damage in the dresser. Under these conditions, the dresser cannot provide the adequate dressed state to the grinding wheel, causing increased grinding forces and faster loss of grain edge sharpness [3]. For this reason, monitoring and control of dresser wear is a very important issue; however, most techniques are based on direct measurement of tool wear, such as optical and laser techniques [4] and touch probe contact methods [5]. Furthermore, direct measurement techniques require the machine to be stopped in order to remove the tool for inspection, resulting in high costs for machine tool downtime and human operator intervention.

On the other hand, indirect measurement techniques have been developed as innovative approaches for manufacturing process monitoring [6]. In particular, impedance-based structural health monitoring (SHM) has emerged for its easy applicability due to the use of low-cost lead zirconate titanate (PZT) piezoelectric transducers and simple instrumentation requirements [7,8,9]. This approach has been employed for different structural damage detection applications in aerospace and civil engineering by utilizing the electromechanical impedance (EMI) measurement system [10,11,12]. Since this technique has emerged, however, impedance-based diagnosis for grinding and dressing operations is still a rare instance and the number of research publications on the topic is very small. An application to dressing operation monitoring based on the EM impedance method was proposed in Part 1 of this work and the damage level suffered by the tool during the dressing operation was verified through representative damage indices. However, the selection of the most appropriate frequency ranges for the damage indices calculation needs to be carried out through analytical approaches or artificial intelligence paradigms, which was not covered by that work (Part 1).

Furthermore, according to [13] the selection of the optimal frequency range is important to ensure high sensitivity to damage detection and good repeatability between the measurements, which avoid the acquisition or storage of any unnecessary data. To this end, several researchers have attempted to realize an optimal damage diagnosis based on smart interface and artificial intelligent techniques [12,13,14]. For instance, in [14] a piezoelectric-based smart interface technique was developed to acquire sensitive impedance signatures from a bolted connection for bolt-loosening detection. A simplified EMI model of a smart interface-embedded bolted connection system was formulated to interpret a mechanistic understanding of the EM impedance signatures under the effect of bolt preload. In addition, finite element modeling of a bolted connection was carried out to show the numerical feasibility of the presented method, and to predetermine the sensitive frequency band of the impedance signatures. At the end, authors observed that: (1) the PZT interface’s sensitive frequency band, predetermined by the numerical simulation, was quite consistent with that measured from the experiment, and (2) a single PZT interface was able to monitor multiple loosened bolts in a connection, thus reducing the number of sensing channels for the impedance monitoring of a large bolted connection. In [13] a formal procedure was proposed to determine the frequency ranges in which the PZT transducers are more sensitive for damage detection. To this purpose, experimental tests were carried out on specimens with different sizes and there was a good match between the theoretical and practical results. Thus, a modified equivalent electromechanical circuit to analyze the sensitivity of the PZT transducer in function of the frequency and mechanical impedance of the host structure was presented by the authors, and then the proposed approach was very useful in selecting the correct frequency range in SHM systems based on the EMI technique. 

In addition, recent studies showed that the implementation of a network of PZTs in the EMI technique instead of a single PZT transducer provides more accurate results concerning the detection and the evaluation of damage in structural members constructed by concrete slabs and reinforced concrete [15,16,17,18,19], and composite slabs [20,21], by using smart interface and numerical modelling to achieve the most sensitive features. On the other hand, in [22] an innovative EMI incorporating an artificial neural network (ANN)-based pattern analysis tool was proposed to identify damage-sensitive frequency ranges autonomously and to provide detailed information, such as the damage type and severity in a simply supported aluminum beam. At the end, the proposed approach efficiently provided damage information for various damage cases with autonomously pre-determined damage-sensitive frequency ranges. In [23], a new technique for autonomous selection of damage-sensitive frequency ranges using ANN based on EMI was proposed. The proposed approach combined all the information from the available data obtained from a wide frequency range and effectively estimates the severity of various damage cases by optimal selection of damage-sensitive frequency ranges. However, the approaches mentioned above for dressing tool condition monitoring have not been attempted yet. Therefore, in this paper, a method based on multi-layer neural network (MLNN) sensorial data processing, integrated with the k-nearest neighbor classifier algorithm (*k*-NN), is proposed for impedance-based dressing tool condition monitoring. MLNNs are powerful computational tools that model the basic behavior of logical thought patterns by numerical representation using artificial neurons linked to each other. The advantage of this type of ANN is given by its flexibility and simplicity of implementation with a straightforward training process [24]. Besides that, the k-nearest neighbor classifier (*k*-NN) algorithm is a well-formulated nonparametric pattern-classification technique widely used for pattern classification problems [25,26]. After the MLNN training process, the *k*-NN algorithm will be used to evaluate the neural network performance in dressing tool health monitoring.

This paper intends to contribute to filling the gap in dressing tool condition monitoring. To this end, experimental dressing tests were performed with two types of dressing tools made of synthetic and natural diamonds, respectively. Two low-cost PZT transducers were mounted in different positions on the dresser holder and an EMI measurement system was employed to detect the impedance signal signatures. As input to the MLNN, dressing tool damage information was extracted by using representative damage indices for various damage cases at different frequency bands. Consequently, the proposed intelligent system is expected to select the most damage-sensitive features based on the optimal frequency band recognized in the validation step by the *k*-NN algorithm.

The main contributions of this research work are summarized in the following two points. First, in the context of Industry 4.0, this approach forms a paradigm shift from traditional dressing tool condition monitoring systems using sensor signals to a new perspective using an alternative and nondestructive evaluation technique integrating low-cost PZT transducers. Second, the effective implementation of the proposed approach for industrial grinding operations can be taken into account in Computer Numerical Control (CNC) machines with low cost and without significant changes in the usual cycle times.

## 2. Dressing Operation Monitoring

Many researchers have made considerable efforts to establish precision monitoring and diagnostic systems for manufacturing processes using indirect measurement methods which have the advantage of not interfering with the process [27]. These methods are typically based on sensor signal detection and processing signals (acoustic emission, vibration, force, power) for the monitoring of tool wear, tool breakage, process conditions, fault detection, etc. [17,18,19]. Monitoring of dressing operations conducted so far follows the same framework reported in the literature [20,21]. However, few studies were focused on the monitoring of dressing tool health in terms of wear, damage and breakage of dressing tools made of diamond [28,29]. Several studies have been focused on grinding wheel health monitoring in order to determine the best moment to carry out or to stop the dressing operation [30,31,32]. Kwak et al. [33] used the grinding force signal based on discrete wavelet decomposition to establish the dressing time. Oliveira et al. [34] presented a method to produce patterns on the wheel surface during the dressing operation based on Acoustic Emission (AE) mapping. Brzezinski et al. [35] proposed a new method for grinder dressing fault mitigation using real-time peak detection. In Junior et al. [36,37], the evaluation of single-point dresser wear was proposed through intelligent spectral analysis of vibration signals. Miranda et al. [38] presented a methodology to estimate the wear of single-point dressers based on AE, vibration and fuzzy models.

A novel technique for dressing metal-bonded diamond grinding wheels using technologies of abrasive waterjet and touch truing was proposed by [39]. Martins et al. [40] described a study to classify the wear of single-point dressers using AE and ANN. In order to identify the tool condition and determine a stopping criterion for the dressing process, Lopes et al. [41] used the power spectral density and event counts of AE. D’Addona et al. [42] proposed a method to characterize the dressing tool utilizing cognitive paradigms.

Furthermore, the cost reduction is still a challenge for these monitoring systems. Conventional techniques present high computational cost because of the several phases of data processing and the systems require expensive instrumentation and sensors. Besides that, the techniques not employing intelligent approaches require the manual selection of the best sensor signal features. Thus, an alternative monitoring system based on electromechanical (EM) impedance is considered in this paper.

## 3. Impedance-Based SHM Principle

### 3.1. Electromechanical Impedance Technique

Among the different SHM techniques, electromechanical impedance (EMI) has emerged as a promising, non-destructive, cost-effective, and highly sensitive solution for real-time damage diagnosis [7]. The transducers, usually lead zirconate titanate (PZT), are glued to the structure to be monitored. Due to the piezoelectric effect, a relationship between the mechanical properties of the structure and the electrical impedance of the transducer is established. This allows variations in these properties to be monitored by measuring the electrical impedance [8]. In the EM impedance method, each transducer allows a forced excitation of the structure and the simultaneous measurement of electrical impedance. Therefore, it is common in the literature to attribute the roles of sensor (direct piezoelectric effect) and of actuator (reverse piezoelectric effect) to PZT ceramics. The relationship between the electrical impedance of the piezoelectric transducer and the mechanical impedance of the structure to be monitored can be determined by means of electromechanical models. In one of the simplest models for a square PZT patch [7], the electrical impedance is given by Equation (1):

(1) $$Z_{E}\left(\omega \right)=\frac{1}{j\omega C_{0}}\|jZ_{T}{\left(\frac{s_{11}}{d_{31}l}\right)}^{2}\left[\frac{1}{2}\tan\left(\frac{kl}{2}\right)-\frac{1}{\sin\left(kl\right)}+\frac{Z_{s}}{j2Z_{T}}\right]$$

where $Z_{E}\left(\omega \right)$ is the electrical impedance, ω is the angular frequency, $C_{0}$ is the static capacitance for a square patch of size l, k is the wave-number, $Z_{T}$ is the mechanical impedance of the piezoelectric patch, $Z_{s}$ is the mechanical impedance of the monitored structure, $d_{31}$ is the piezoelectric constant, $s_{11}$ is the compliance at a constant electric field, ∥ indicates a parallel connection, and j is the unit imaginary number.

### 3.2. Representative Damage Indices

In SHM analysis, damage is identified by comparing the measured electrical impedance in an initial condition (healthy), with the impedance measured after the structure underwent damage. This comparison is made by means of damage metrics, such as the root mean square deviation (RMSD) and the correlation coefficient deviation metric (CCDM). The RMSD is based on Euclidean distance between two impedance signatures, as given by Equation (2), which was used in [43]. The CCDM is based on the correlation coefficient between the two impedance signatures, as given by Equation (3), respectively [7,8].

(2) $$RMSD={\left(\sum_{k=\omega_{l}}^{\omega F}{\left[Z_{E,D}\left(k\right)-Z_{E,H}\left(k\right)\right]}^{2}/\sum_{k=\omega_{l}}^{\omega F}{\left[Z_{E,H}\left(k\right)\right]}^{2}\right)}^{1/2}$$

(3) $$CCDM=1-\frac{\sum_{k=\omega_{l}}^{\omega F}\left[Z_{E,H}\left(k\right)-{\bar{Z}}_{E,H}\right]\left[Z_{E,D}\left(k\right)-{\bar{Z}}_{E,D}\right]}{\sqrt{\sum_{k=\omega_{l}}^{\omega F}{\left[Z_{E,H}\left(k\right)-{\bar{Z}}_{E,H}\right]}^{2}}\sqrt{\sum_{k=\omega_{l}}^{\omega F}{\left[Z_{E,D}\left(k\right)-{\bar{Z}}_{E,D}\right]}^{2}}}$$

where the subscripts H and D indicate the healthy and damaged conditions, respectively, while $Z_{E,H}$(*k*) and $Z_{E,D}$(*k*) are the electrical impedance signatures (i.e., the magnitude, the real part or the imaginary part) of the structure under intact and damaged conditions, respectively, which are measured at a frequency k that ranges from ω1 (the initial frequency) to *ωF* (the final frequency). ${\bar{Z}}_{E,H}$ and ${\bar{Z}}_{E,D}$ are the averaged signatures under healthy and damaged conditions [7].

## 4. Multi-Layer Neural Networks and K-Nearest Neighbor Algorithm

### 4.1. MLNN Principle and Training Algorithm

In this paper, the use of multi-layer neural networks (MLNN) that compute the output directly from the input in one iteration based on a feedforward network [44], was proposed. Nowadays, the use of a multi-layer perceptron to implement a MLNN is the most common [24]. A single-layer perceptron can produce only linearly separable decision regions. For this reason, a MLNN consists of three or more layers (i.e., an input layer, one or more hidden layers and an output layer). Therefore, the outputs of the hidden nodes are calculated by Equation (4) and the outputs of the ANN are calculated by Equation (5), respectively [45].

(4) $$z_{j}=f\left(\overset{N-1}{\underset{i=0}{\sum }}w_{ij}^{\prime }x_{i}-\theta_{j}^{\prime }\right)\;for\;j=0,1\ldots ,M-1$$

(5) $$y_{k}=f\left(\overset{N-1}{\underset{i=0}{\sum }}w_{jk}z_{j}-\theta_{k}\right)\;for\;k=0,1\ldots ,L-1$$

where $x_{i}$ are the inputs of the ANN, f is the nonlinearity, $z_{j}$ are the outputs of the hidden layer, $w_{ij}^{\prime }$ the weights between the input and hidden layer, $\theta_{j}^{\prime }$ are internal offsets in the hidden nodes, $w_{jk}$ are the weights between the hidden and output layer and $\theta_{k}$ the internal offsets in the output layer.

In this paper, the inputs $x_{i}$ to the MLNN are given by damage metrics values calculated for N different frequency ranges: (RMSD, i=1…,N) and (CCDM, *i* = 1…*N*).

The backpropagation (BP) algorithm with the Levenberg–Marquardt (LM) optimization, widely reported for feedforward neural network training, was used in this paper. Firstly, the mean square error (MSE) cost function is defined by Equation (6), for a MLNN with K outputs and a set of P training patterns [44].

(6) $$E\left(w\right)=\frac{1}{2}\overset{P}{\underset{p=1}{\sum }}\overset{K}{\underset{i=1}{\sum }}{\left(d_{i}^{\left(p\right)}-y_{i}^{\left(p\right)}\right)}^{2}$$

where $y_{i}^{\left(p\right)}$ and $d_{i}^{\left(p\right)}$ are the output activations and desired responses respectively, and w is the column vector containing all the weights and thresholds of the network. Secondly, the local approximation of the cost function is calculated by a second order method for a quadratic form, given by Equation (7) [44].

(7) $$E\left(w_{k}+dw_{k}\right)=E\left(w_{k}\right)+\nabla E{\left(w_{k}\right)}^{T}dw_{k}+\frac{1}{2}dw_{k}^{T}\nabla^{2}E\left(w_{k}\right)dw_{k}$$

where $\nabla E\left(w_{k}\right)$ and $\nabla^{2}E\left(w_{k}\right)$ denote the gradient vector and the Hessian matrix (also referred to as H) of the cost function, respectively. The Newton step $dw_{k}$ is obtained by the first optimal condition [45] and is given by Equation (8), and the Hessian matrix H is given by Equation (9), respectively.

(8) $$dw_{k}=-{\left[\nabla^{2}E\left(w_{k}\right)\right]}^{-1}\nabla E\left(w_{k}\right)$$

(9) $$H=\nabla^{2}E\left(w_{k}\right)=\left(J_{k}^{T}J_{k}+S_{k}\right)$$

where $J_{k}$ is the Jacobian matrix of first derivatives of the residuals $d_{i}^{\left(p\right)}-y_{i}^{\left(p\right)}$, as it was given by Equation (7). $S_{k}$ is the second order derivative information in $\nabla^{2}E\left(w_{k}\right)$ or H [44]. The Jacobian matrix $J_{k}$ was introduced to simplify the calculating process. Thus, the Levenberg–Marquardt algorithm was designed to approach second-order training speed without having to compute the Hessian matrix H. Therefore, the Levenberg–Marquardt algorithm uses an approximation to the Hessian matrix given by Equation (10) in the following Newton-like update, given by Equation (11).

(10) $$H\approx J_{k}^{T}J_{k}+\mu I$$

(11) $$W_{K+1}=W_{k}-{\left(J_{k}^{T}J_{k}+\mu I\right)}^{-1}\;\;J_{k}e_{k}$$

where μ is always positive, combination coefficient I is the identity matrix. The weights for the whole network, denoted by vector w, store information and are learnt during the training process. The subscript k denotes the iteration step [44].

### 4.2. K-NN Algorithm-Based Approach

In this paper, the k-NN algorithm was used to validate the efficiency of the MLNN approach. The principle is based on the allocation of new unclassified examples to the class to which the majority of its K nearest neighbors belongs. Then, given a test data x, the algorithm finds the K nearest neighbors of x between all the training data, and score the category candidates based on the category of K neighbors. The similarity of x and each neighbor data is the score of the category of the neighbor data [46]. Therefore, if several of the K nearest neighbor data belong to the same category, the sum of the score of that category is the similarity score of the category concerning the test data x. According to [25], the case of ‘the nearest-neighbor rule’ is considerably simpler than the general K case. The nearest-neighbor rule is adopted in this paper and the decision rule of k-NN is given by Equation (12):

(12) $$f\left(x,C_{j}\right)=\underset{t_{i}\in kNN}{\sum }sim\left(x,t_{i}\right)Z\left(t_{i},C_{j}\right)$$

where, input data x is the separated database to be tested, which gets assigned $\left(x,C_{j}\right)$ to the class with the largest probability $C_{j}$, with respect to x. $sim\left(x,t_{i}\right)$ denotes the similarity between x and the database trained by the network proposed in this paper, $t_{i}$. $Z\left(t_{i},C_{j}\right)\in \left\{0,\;1,2,3,\;4,5\right\}$ is the category value of the training data, $t_{i}$, with respect to $C_{j}$ [25]. 

## 5. Experimental Test and Evaluation

Two experimental dressing tests were conducted on a SULMECANICA RAPH 1055 surface-grinding machine, using a conventional NORTON 38A150L6VH aluminum oxide grinding wheel (355.6 × 25.4 × 127 mm). A complete specification of the grinding machine, dressing conditions and test facilities can be found in Table 1.

**Figure 1:**
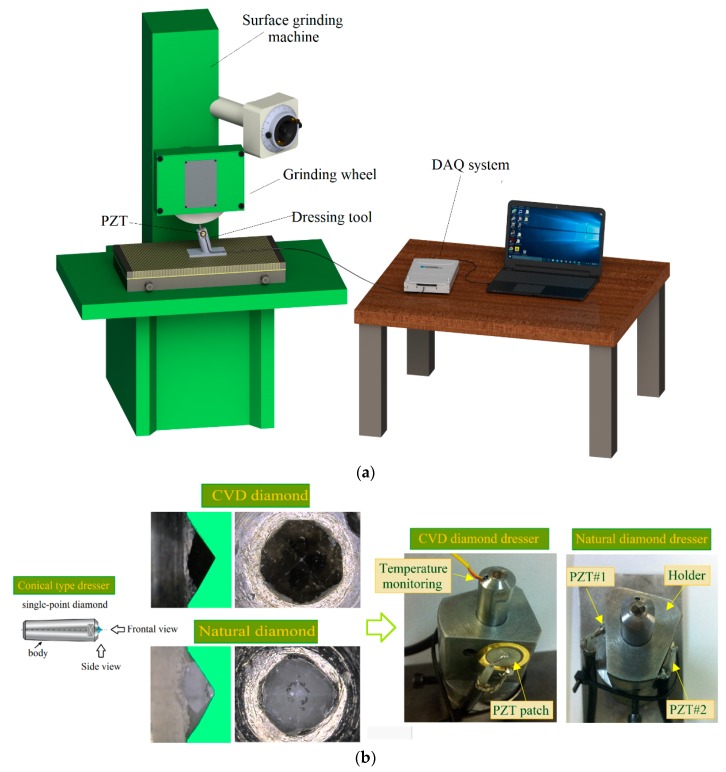
Proposed sensing setup for dressing tool condition monitoring (Part 2): (**a**) schematic representation of the test bench; and (**b**) the two types of diamond dressing tools and the two lead zirconate titanate (PZT) transducers for experimental testing; configuration of the dresser body; top and side views of the diamonds. (DAQ: multifunctional data acquisition; CVD: chemical vapor deposition)

Figure 1a presents a schematic diagram of the test bench. In addition, to evaluate the effectiveness of the proposed approach for dressing tool condition monitoring, two industrial conical type dressers with well-defined diamond geometry were used, as shown in Figure 1b. Figure 1b also presents the top and side views of the dressing tool diamonds before the experiments. Both experiments were conducted without application of cutting fluid, i.e., in dry-dressing conditions, similar to that used in [47], since, under these conditions, wear is accelerated, allowing a reasonable timeframe of experimentation.

In relation to single-point dressing parameters adopted in this study, it is accepted in literature that one important parameter in the dressing process is the overlap ration (*U_d_*), which indicates the relationship between the width of action (*b_d_*) of the dresser diamond and the dressing feed rate (*f_d_*). In this case, *b_d_* is given by a dressing depth (*a_d_*). These parameters are directly connected with the sharpness that the dressing operation produces on the grinding wheel. In this study, an *U_d_* = 1 was adopted at the beginning of the experiment, according to literature recommendations [3,5,36]. The other main parameters were the constant dressing depth equal to 40 µm and the constant dressing speed set at 3.45 mm/s.

Additionally, an experimental analysis of the wear state of the diamond dressing-tool based on [5] was conducted on a TESA-VISIO-200 microscope, with 6.5× optical magnification (0.7× to 4.5×), equipped with 200 × 100 mm measuring table and a TESA-REFLEX interface software. The macro images of the diamond tips were captured for both top and side views, and the magnification of 0.7× was adopted.

Two PZT transducers MURATA 7BB-20-6 [48] diaphragm type were used as shown in Figure 1b. This type of transducers is constructed of a circular bronze disc with a diameter of 20 mm (chosen size) and thickness of 0.20 mm, also a piezoelectric ceramic (active element) with a diameter of 14 mm and thickness of 0.22 mm. These two patches were attached on the dresser holder in opposite equidistant directions for comparison purpose. In this paper, an alternative system proposed by Baptista and Vieira Filho [8] was used to measure the electrical impedance. It is based on a multifunctional data acquisition (DAQ) device controlled by a computer equipped with an appropriate software and drivers for interface. The basic principle of this system was related in Part 1 of this work. A NATIONAL NI USB-6221 DAQ device and a computer equipped with LABVIEW software were used to acquire the signatures from two PZT transducers simultaneously, at sampling rate of 250 kS/s. The transducers were excited through a chirp signal with magnitude of 1 V and frequency range from 0 to 125 kHz, also a resistor of 2.2 kΩ was used.

During the experiments, temperatures at the diamond tip were kept at 30 °C constant at the impedance measurements in accordance with [49]. A MINIPA MT455 digital thermometer equipped with a type K thermocouple was used to measure the temperature. The thermocouple was fixed in the dresser body through a hole in the diamond region, where the thermal conductivity is high, i.e., 1000 W/(mK) (e.g., steel is 50.2 W/(mK)) [5,47].

### 5.1. Experimental Test Case # 1: CVD Diamond Dressing Tool

#### 5.1.1. Procedure Description

Dressing tests with the above configuration were carried out with a single-point dresser made of chemical vapor deposition (CVD) diamond, as shown in Figure 1a. Each test consisted of dressing passes throughout the grinding wheel surface until the end of the grinding wheel lifespan. This experiment with the CVD diamond dresser involved as many as 600 dressing passes, and three damage conditions defined every 200 dressing passes (damage 1 (D1), damage 2 (D2), damage 3 (D3)) were compared with the baseline signal from the healthy CVD diamond dressing tool (H), associated with the CVD diamond dressing tool before the tests.

**Figure 2:**
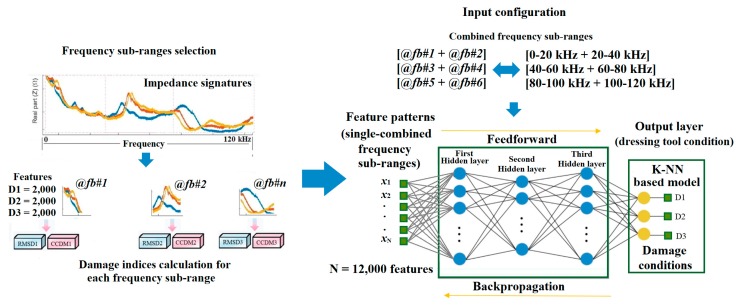
Intelligent dressing tool health monitoring approach based on electromechanical impedance (EMI).

As is well known, the impedance signatures are sensitive to minor changes in the host structure. In addition, to obtain the impedance signatures sensitive to the damage, the measured frequency range should contain those resonant frequencies related to the monitored structure [14]. Therefore, to analyze the results of this study, both damage metrics RMSD and CCDM were calculated for several frequency bands from 0 to 125 kHz. This frequency range was selected due to the sampling rate of the DAQ device used in this research work (i.e., 250 kS/s). The real part of the impedance was considered for this purpose. According to Table 2, six frequency sub-ranges were defined within a wide frequency band of 0–120 kHz, with each frequency sub-range equal to 20 kHz. The choice of the frequency sub-range value was optimally selected for the present application based on experimental impedance signatures and on the values obtained from the damage metrics, as well as following the approaches presented in [7,50]. The sampling rate for damage indices calculation was 10 Hz. In this way, for a given sensor, for a given damage metric and for a given frequency sub-range, the training inputs to the ANN are made of 2000 features for each of the three damage conditions (D1, D2, D3), i.e., they consist of input patterns each containing 6000 features with each feature related to only one of the three damage conditions: D1 or D2 or D3. As two transducers were utilized (PZT#1, PZT#2), two damage metrics were calculated (RMSD, CCDM) and six frequency sub-ranges were considered (0–20 kHz or 20–40kHz or 40–60 kHz or 60–80 kHz or 80–100 kHz or 100–120 kHz), a total of 2 × 2 × 6 = 24 training patterns were constructed for supervised learning of 24 ANN models. In addition, a study was conducted to identify the best ANN architecture. Each ANN model was trained and tested by combining the input patterns of two consecutive frequency sub-ranges, based on [37,40], to yield a combined input pattern containing 12,000 features for each of the three resulting combined frequency sub-ranges: [0–20 kHz + 20–40 kHz], [40–60 kHz + 60–80 kHz], [80–100 kHz + 100–120 kHz] (Table 3). The scope was to find the best frequency sub-range combination capable to provide the reliable monitoring of dressing tool condition. This procedure was adopted following the approach presented in [12]. Furthermore, a schematic representation of the training set construction as well as the ANN structure is shown in Figure 2.

**Figure 3:**
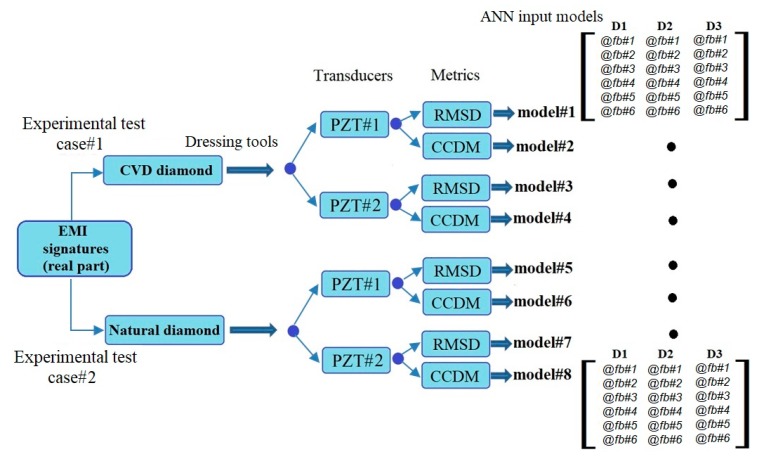
Artificial neural network (ANN) model construction.

Figure 3 illustrates the construction of the ANN models. Four ANN models were constructed for the experimental test case 1 (*model#1*, *model#2*, *model#3*, and *model#4*). A binary matrix that considered the three damage conditions D1, D2 and D3 was built as target for the ANN model output. A logic value 1 was used to activate the features corresponding to each damage condition D1, D2, D3. Multi-layer perceptron network architectures with 1 to 3 hidden layers were created, varying the number of neurons from 5 to 40 per layer. Supervised ANN models with Levenberg–Marquardt (LVM) backpropagation function were implemented and trained using MATLAB software. To evaluate the repeatability of the proposed configuration, each ANN model was trained five times for each set of input patters and 10% of the features were kept aside for validation. The performance evaluation for the 10% of the validation features that were not used in the training stage was carried out through the k-NN algorithm to classify the dressing tool damage condition. To this end, 200 features for each damage condition (10% of 2000 features) were considered and a standard implementation case was chosen with k=1 (nearest-neighbor rule), which is considered a simplified case in terms of computational processing and implementation [46].

#### 5.1.2. Results and Discussion on the Analysis of the Wear State of Diamond Tool

**Figure 4:**
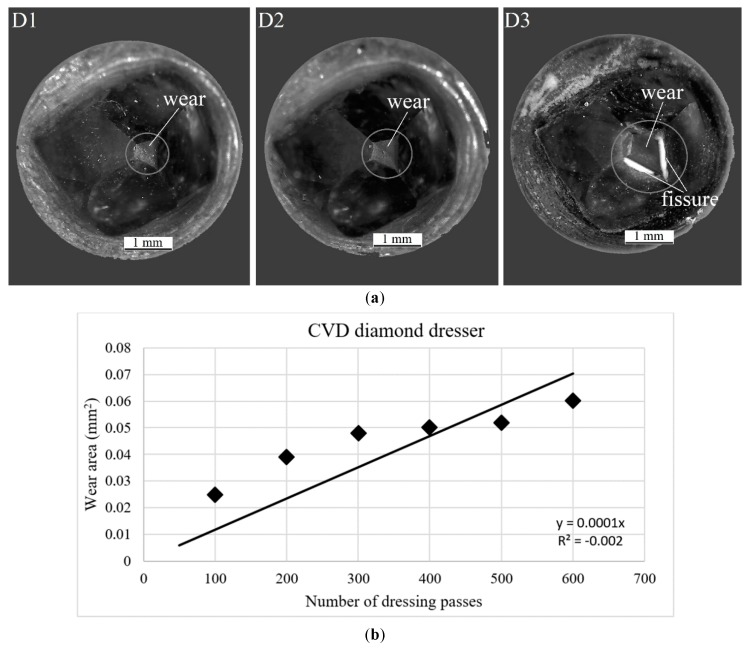

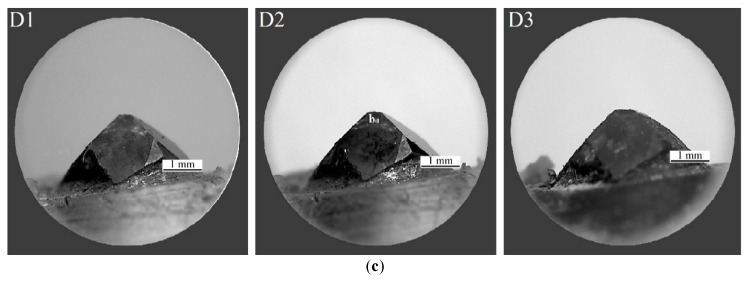
Analysis of the CVD diamond wear state: (**a**) top view; (**b**) wear area trend; and (**c**) side view.

Considering that the purpose of the work was to find a correlation between the damage state of the CVD diamond dressing tool and the impedance signature, Figure 4 shows micrographs of the CVD diamond dressing tool after 200, 400 and 600 dressing passes, respectively, as well as the wear development of the CVD diamond dressing tool as a function of the number of dressing passes.

The industrial diamond manufacturing process is characterized by stochastic components and depends on various factors, such as the impurities in natural diamonds, the methods to produce CVD diamonds, the metallization and characterization processes. These effects are well covered in the literature (see for instance [51]). For this reason, even though two diamonds with the same reference and under the same dressing conditions are used in this study, the number of passes for the CVD diamond dresser differs from the number of passes for the natural diamond dresser (see Section 5.2—Experimental Test Case # 2: Natural Diamond Dressing tool). For the CVD diamond, it was verified that this material presented such a high wear resistance that it was not possible to reach the end of its life, since the grinding wheel life was over before the end of dresser tool life due to excessive reduction of grinding wheel diameter. Figure 4a shows that the condition of the diamond did not change significantly with increasing number of passes: it is possible to observe only a small wear and some fissures at 600 dressing passes in the top views. Moreover, in the side views (Figure 4c), for a value of overlap ration *U_d_* = 1 at the beginning of the dressing and for constant values of dressing depth, *a_d_*, and dressing feed rate*, f_d_*, the width of action, *b_d_*, does not suffer considerable changes that could increase the contact area between grinding wheel and diamond, directly influencing the final quality of the dressing and grinding process.

Figure 4b shows the evolution of the CVD diamond tip wear. The side worn area of the CVD diamond tip was measured every 100 dressing passes using NX CAD software and plotted vs. the number of passes showing an increasing trend of the wear development. However, this trend was not exactly linear as the dressing passes took place. Thus, in accordance with previous observations by microscopy photographs, the effects of the high wear resistance presented by this material was also evident in Figure 4b, since the worn area increased at the beginning (100, 200, and 300 passes, respectively), and then, after 300 dressing passes it did not present significant increases up to 500 passes, i.e., it kept similar values at approximately 0.05 mm^2^. After 500 passes, it increased again up to 600 passes. The CVD diamond tip presented less than 10% of worn area for 600 passes due its very high wear resistance (loss of 0.06 mm^2^). This result is in agreement with previous research work, such as [37,40], where authors addressed wear condition assessment of single-point dresser by studying the frequency content of acoustic emission and vibration signals for several frequency bands. Neural network models were applied to classify the diamond wear during dressing with satisfactory results although high standard deviations were observed, probably due to different friability properties of the utilized diamonds.

#### 5.1.3. Results and Discussion of the MLNN Performance Assessment: Confusion Matrix and k-NN-Based Decision Boundary Graphic

This section presents the validation process following the training performed with the MLNN using the LVM backpropagation algorithm for each ANN model constructed. The main characteristics of each model are presented in Table 3. In order to identify the best frequency band to be utilized, an average error was computed for each combined frequency sub-range considered for the generation of the 12,000 features combined input pattern to the ANN model. Table 3 shows that the combined input pattern related to the combined frequency sub-ranges [*@fb#3* + *@fb#4*], corresponding to [40–60 kHz + 60–80 kHz], presented the lowest overall testing error for CVD diamond dressing tool damage classification. This combined input pattern presented a high percentage of testing errors for some models, in particular *model#2* and *model#4.* However, when compared with the other two combined frequency sub-ranges, this combined input pattern provides a much better accuracy. It is worth mentioning that the other two combined input patterns presented errors higher than 10% for some models, in particular *model #2* and *#3* for combined input pattern [*@fb#1* + *@fb#2*], and model#3 for combined input pattern [*@fb#5* + *@fb#6*] (Table 3). On the other hand, the errors of the combined input pattern related to the combined frequency sub-ranges [*@fb#3* + *@fb#4*] were lower by 10% for all models, which is an interesting result for real-time monitoring implementation. In summary, this combined input pattern presented an average error of 3.2%, against 9.1% and 6.5% for the other two combined input patterns, and its standard deviation was 2.6%, against 8.1% and 5% for the other two combined input patterns.

From Table 3, it can be seen that the best ANN models for the combined input pattern related to the combined frequency ranges [40–60 kHz + 60–80 kHz] were model#1 (using PZT#1 sensor and RMSD metric) and model#3 (using PZT#2 sensor and RMSD metric) presenting errors of 1.1% and 0.9%, respectively. Furthermore, Table 4 shows the main characteristics resulting from the two best ANN models identified during the validation phase. The first column shows the considered parameters in the feature extraction process: combined input patterns, neurons structure, training function and *k*-NN rule for dressing tool health features classification. The second column reports the specifications for each parameter. According to the results, both model#1 and model#3 presented a simple structure with three hidden layers of 10-10-5 neurons and two hidden layers of 5-15 neurons, respectively, which is very attractive in terms of implementation effectiveness.

**Figure 5:**
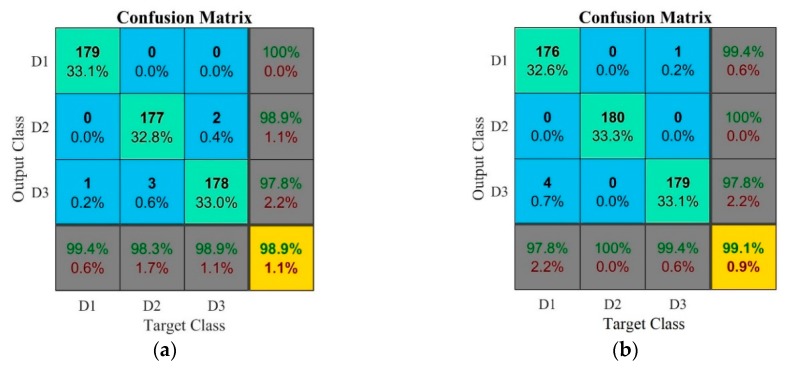
Confusion matrices of the best ANN models in experimental case #1: (**a**) *model#1*; and (**b**) *model#3*.

**Figure 6:**
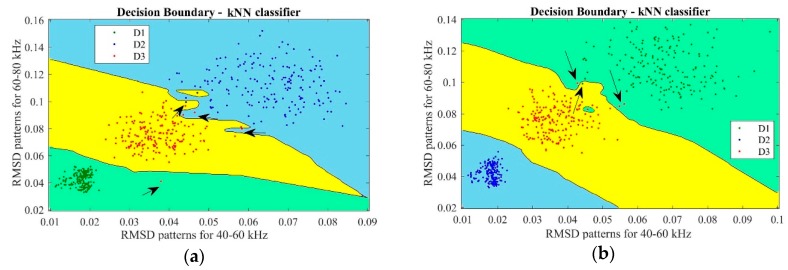
*k*-NN results via decision boundary graphics for the best ANN models in experimental case #1: (**a**) *model#1*; and (**b**) *model#3*.

Further comparisons and analyses are illustrated in Figure 5 and Figure 6, where confusion matrices and decision boundary graphics present the best ANN performances for experimental test case 1. Confusion matrices are often used to summarize the performance of a classification model [40]. Accordingly, the results of *model#1* and *model#3* are presented in Figure 5a,b, respectively. As regards *model#1* (Figure 5a), for the D1 damage condition the ANN presented a predictive capability of 100% and a sensitivity of 99.4%. For the D2 damage condition, the predictive capability was 98.9% and the sensitivity was 98.3%, corresponding to the occurrence of false positive diagnostics. For the D3 damage condition, the confusion matrix showed a predictive capability of 97.8% and a sensitivity of 98.9%. The overall accuracy rate of the ANN for *model#1* was 98.9%, which is attractive for actual implementation.

Figure 5b presents the results of model#3: the overall accuracy rate of the ANN for this model was 99.1%, i.e., the network was able to accurately distinguish the data at this level of dresser damage and the patterns were classified correctly. For the D1 damage condition, the ANN presented a good performance: 99.4% and 97.8% for predictive capacity and sensitivity parameters, respectively. For the D2 damage condition, both predictive capability and sensitivity parameters were 100%. For the D3 damage condition, the predictive capacity was 97.8%, corresponding to false negatives, and the sensitivity was 99.4%. For this reason, this model is very attractive in terms of practical implementation for dressing tool heath monitoring. This result agrees with the findings in [40], where most of the best neural network models, with small overall classification error, also presented false negatives for the worn dresser condition (e.g., the ANN predicted that the dresser was in half-life condition whereas it was fully worn instead).

Figure 6 reports the decision boundary graphics based on *k*-NN classifier for the best models achieved by ANN for the experimental case 1, according to Table 4. Boundary regions graphic is a visual way to analyze the results of the *k*-NN procedure; this approach is based on an over fitting process, according to [52]. In this kind of graph, the location of each point refers to the real damage condition, while its color shows the classification performed by the *k*-NN based on successful training of the ANN. In Figure 6, the arrows indicate the features that have not been classified correctly. Since model#1 and *model#3* presented good results, the classification performance is very high with only a small number of features misclassified in the *k*-NN procedure. In Figure 6a (*model#*1), it can be observed that some features belonging to the D3 damage class were classified as D1 and D2. On the other hand, only one feature belonging to the D2 damage class was classified as D3. In Figure 6b (model#3), it is possible to see that one feature belonging to the D1 damage class was classified as D3, as well as some features belonging to D3 were classified as D2. This result again agrees with the literature, since most of the damage features erroneously classified occurred in regions close to the borders, as reported in [37] where the authors did not consider them as serious errors because these boundaries can be difficult to determine.

### 5.2. Experimental Test Case # 2: Natural Diamond Dressing Tool

#### 5.2.1. Procedure Description

A second series of dressing tests were carried out, under the same dressing conditions as for experimental test case 1, using a stationary single-point dressing tool made of natural diamond. For structural damage detection, the impedance measurements were performed at dressing pass intervals varied according to the test conditions, with the aim to detect variations in the impedance of the monitored structure (diamond dressing tool) for different damage conditions. In experimental test case 2, the testing consisted of dressing passes throughout the grinding wheel surface till the end of the natural diamond tool life which involved 300 dressing passes. Again, three damage conditions were defined after every 100 dressing passes (damage 1 (D1), damage 2 (D2) and damage 3 (D3)) which were compared with the baseline signal corresponding to the healthy natural diamond dressing tool (H). The results of the impedance measurements, as well as the ANN architectures in terms of training and validation phases, were carried out using the same procedures as for experimental test case 1 (Figure 2 and Figure 3). Accordingly, the same six frequency sub-ranges reported in Table 2 were considered also for experimental test case 2. Therefore, those other four models showed in Figure 3 (*model#5*, *model#6*, *model#7*, and *model#8*) were considered.

#### 5.2.2. Results and Discussion on the Analysis of the Diamond Wear State

**Figure 7:**
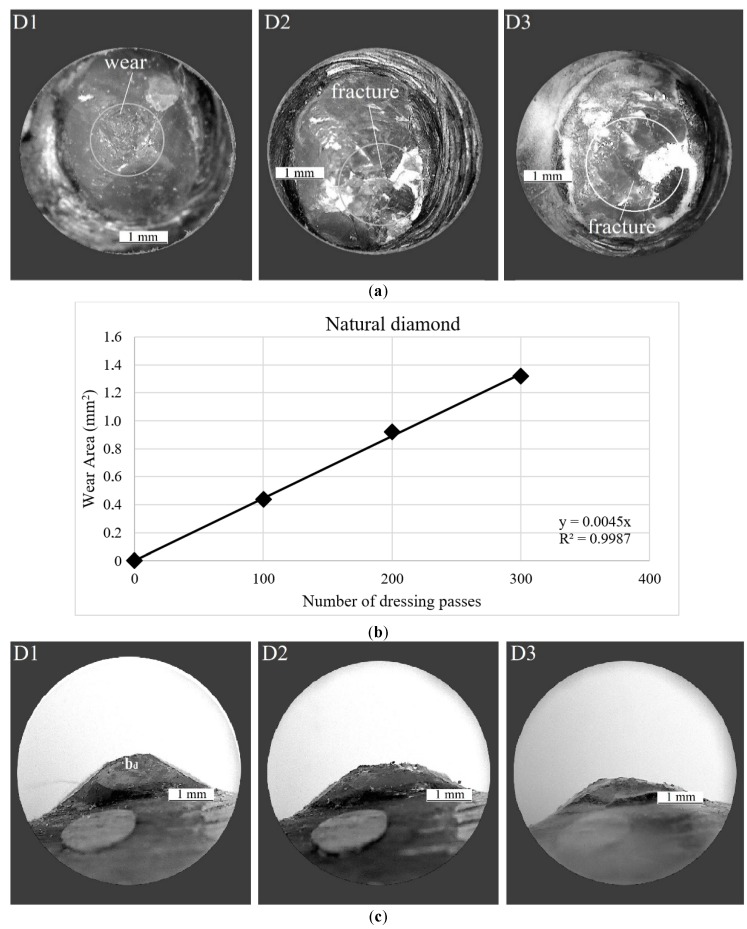
Analysis of the natural diamond wear state: (**a**) top view; (**b**) wear area trend; and (**c**) side view.

As expected, a clear difference is evidenced when comparing the results obtained when using different dressing tool diamonds. Figure 7 shows microscopic photographs of the natural diamond dressing tool after 100, 200 and 300 dressing passes, respectively, as well as the development of natural diamond dressing tool wear as a function of the number of dressing passes.

A significant difference of the diamond conditions can be observed as the dressing passes progress, displaying a much lower wear resistance than for the CVD diamond dressing tool. From Figure 7a,c, it can be observed that the condition of the diamond changed significantly as the dressing passes increased. By examining the top and side views of the natural diamond tool, the wear level after 100 dressing passes is already quite evident and severe damage is verified after 200 and 300 dressing passes evidencing excessive wear and tool fractures. In this case, the increase of the contact area between grinding wheel and diamond tool was higher with a significant growth of the width of action, *b_d_*, parameter for a constant dressing depth, *a_d_* = 40 µm, and a constant dressing feed, *f_d_* = 3.45 mm/s. 

Figure 7b shows the evolution of the natural diamond tip wear that was measured after every 100 dressing passes. The wear area of the natural diamond tip increased as the dressing passes took place, as expected. In contrast with the evolution of the CVD diamond tip wear, presented in Section 5.1.2, a linear trend is observed in Figure 7b. Accordingly, it was possible to determine the actual state of the dressing tool and predict its influence on the grinding operation. In fact, up to 100 dressing passes, *U_d_* increased and, consequently, the sharpness that the dressing operation produces on the grinding wheel also increased. However, after 200 dressing passes, the contact surface shows a dramatic increase, and it grows constantly up to 300 dressing passes. Thus, the behavior of the dressing tool becomes erratic due to the occurrence of fractures and micro-cracks, i.e., catastrophic failures of the diamond dressing tool structure at 300 dressing passes. In accordance with previous observations, the properties of the diamond tip were also evident during the experimental test case #2, since, in contrast with the CVD diamond tip results previously shown in Section 5.1.2, the natural diamond tip was largely affected by the increase of dressing passes. It is worth noting that in [5] this fact was considered as evidence that the diamond wear progressively increased the actual contact area between grinding wheel and diamond tool which lead to higher power consumption during dressing. In addition, the effect of wear in the dressing tool is also evident in the present work: in Figure 7b the natural diamond presented 100% of worn area at 300 dressing passes (1.319 mm^2^). As indicated by the R^2^ = 0.9987 for y = 0.0045x, a proportional trend between the number of the dressing passes and the dressing tool wear level was found. Such results showed that the structure of the natural diamond dresser can have a strong influence on the dressing results as well as on the dressing tool life. This result agrees with previous results obtained in [26,29] where the diamond wear increase with increasing number of dressing passes was clearly evidenced.

#### 5.2.3. Results and Discussion of the MLNN Performance Assessment: Confusion Matrix and k-NN-Based Decision Boundary Graphic

This section presents the results obtained from the ANN models for experimental test case # 2 with the natural diamond dressing tool. The main characteristics of each ANN model are reported in Table 5, following the same characteristics previously adopted for experimental test case 1 (Table 3). From Table 5, it can be seen that the combined input pattern related to frequency sub-ranges [*@fb#3* + *@fb#4*], corresponding to [40–60 kHz + 60–80 kHz], again presented the lowest overall error equal to 2.8% against 12.8% and 5.8% for the other two combined frequency sub-ranges. Again, this combined input pattern presented a high percentage of testing errors for some models, such as *model#6* and *model#8*. However, when compared with the other two combined frequency sub-ranges, its performance was much better. It is worth mentioning that, according to Table 5, again the other two combined input patterns displayed testing errors >10% for some models, which is not attractive in terms of accuracy for actual implementation. Besides this, in experimental test case 2 the testing errors of the best combined input pattern were less than 10%, and the ANN models presented a much better accuracy than for all other approaches, which is very interesting for real-time system monitoring implementation.

It is clear from Table 5 that the best models for the combined input pattern related to the combined frequency sub-ranges [40–60 kHz and 60–80 kHz] were *model#5* (using PZT#1 sensor and RMSD metric) and *model#7* (using PZT#2 sensor and RMSD metric) with testing errors 1.1% and 0.9%, respectively. This means that the RMSD metric presented better results than the CCDM metric for both experimental test cases. Table 6 reports the main characteristics for both best models identified in the validation step, following the same characteristics previously adopted for experimental test case 1. According to the results, both *model#5* and *model#7* presented a simple structure with three and two hidden layers with 5-5-5 and 5-15 neurons, respectively, which again is very attractive in view of implementation. Accordingly, these results are consistent with the previously reported results for experimental test case 1 and confirm the validity of the proposed approach for a broader situation.

**Figure 8:**
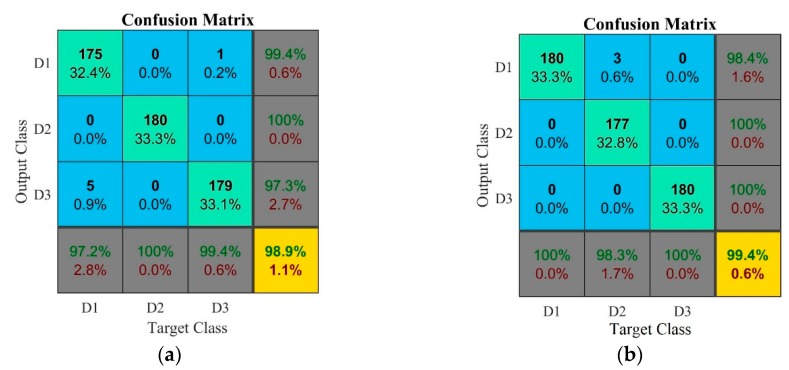
Confusion matrices of the best ANN models in experimental case #2: (**a**) *model#5*; and (**b**) *model#7*.

**Figure 9:**
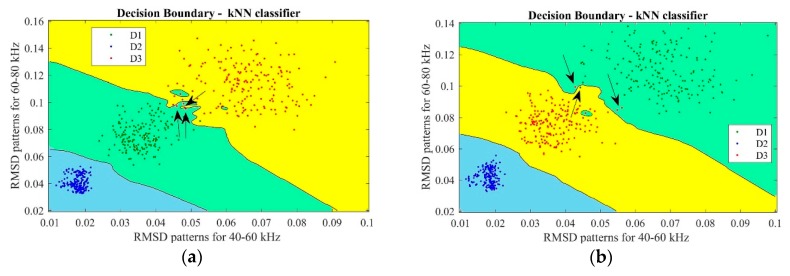
*k*-NN results via decision boundary graphics for the best ANN models in experimental case #2: (**a**) *model#5*; and (**b**) *model#7*.

In Figure 8 and Figure 9, respectively, confusion matrices and decision boundary graphics present the best ANN performance for experimental test case 2. Figure 8a shows the confusion matrix for model#5, using PZT#1 sensor and RMSD metric. For damage condition D1, the predictive capability was 99.4%, corresponding to the occurrence of only one false positive, and the sensitivity was 97.2%. For damage condition D2, the ANN presented a very high performance with 100% for both predictive capability and sensitivity. For damage condition D3, the sensitivity was 99.4% and the predictive capability was 97.3%. The overall accuracy rate of the ANN for this combined input pattern was 98.9%, which is a decidedly attractive result for dressing tool condition monitoring.

Figure 8b shows the confusion matrix for *model#7*, using PZT#2 sensor and RMSD metric. The results of this ANN model showed the best performance of all cases presented in this paper as its overall accuracy rate was 99.4%, i.e., very close to 100%, with only 0.6% of testing error (Table 5). For this reason, this ANN model is very attractive for actual implementation at low-cost in industrial grinding processes. For damage condition D1, the ANN presented 98.4% of predictive capability and 100% of sensitivity. For damage condition D2, the predictive capability was 100% and the sensitivity was 98.3%. For damage condition D3, both sensitivity and predictive capability were 100%. For experimental test case #2, the results show that the proposed approach provides for a more robust diagnosis as the system was able to diagnose the catastrophic tool failure that the natural diamond dresser suffers at 300 dressing passes. This result is in agreement with the results obtained for experimental test case 1, where the overall accuracy rates were similar and close to 100%. In [40] an ANN model that presented 100% success rate in the classification of diamond tool wear level based on the analysis of acoustic emission signals and the combination of various ANN models and several frequency bands. However, the ANN model had only two levels of classification and utilized only one input.

## 6. Conclusions

Due to its notable potential for damage diagnosis, the EMI technique can provide an actual breakthrough in the research on sensor-based grinding process monitoring. This approach was applied for dressing tool health monitoring, incorporating an intelligent decision-making system based on MLNN and *k*-NN algorithm. Two experimental test cases were carried out by fixing two types of stationary single-point dressing tools made of diamond materials: CVD and natural diamonds. During the experiments, sensorial information on the dressing tool was analyzed by considering the real part of the impedance signatures that were obtained from two PZT diaphragms mounted on the dressing tool holder. Microscopic analyses of the diamond tool wear state were applied to quantify the tool wear increase with increasing number of dressing passes. The results indicated that the diamond structure type had a remarkable influence on the dressing process quality and dressing tool life. It is worth noting that during the ANN training phase, representative features were learnt directly from dressing tool health information obtained from RMSD and CCDM metrics with reference to six frequency bands carefully selected. The proposed approach was able to realize a robust dressing tool health monitoring based on the optimal frequency bands identified in the validation phase. The most sensitive combined frequency sub-ranges [40–60 kHz + 60–80 kHz] showed less than 2% of general average error in determining the health patterns of the dressing tools through the three damage classes D1, D2 and D3. The best models showed overall testing errors of 0.6%, 0.9% and 1.1%, respectively, considering the RMSD metric which was more sensitive than the CCDM metric for both experimental test cases. In addition, the results showed that the proposed approach provides high diagnosis accuracy for dressing tool health monitoring and opens the door for its wide application in industrial grinding operations. Accordingly, the effective implementation of the proposed approach for grinding processes in industry can be taken into account in CNC machines at low cost and without significant changes in the usual cycle times due to the simplicity of the EMI technique and its inexpensive PZT transducers. It is worth mentioning that the optimal frequency sub-ranges identified can be applied only to the experimental conditions covered in this paper, since the sensitivity of PZT transducers to select the appropriate frequency ranges for EMI-based SHM systems is a critical issue that always depends on specific factors, such as the properties of each structure and type of damage to be detected. However, the attention is given to the proposed technique, which can be applied for frequency ranges different from those considered in this study. Future research work will entail deeper studies of the dressing tool configuration to include stationary multi-point and rotary dressing tools and to expand the effectiveness and reliability of the proposed approach.

## Figures and Tables

**Table 1:** Experimental test specification.

Parameter	Specification
Grinding machine	SULMECANICA RAPH 1055 surface-grinding machine
Grinding wheel	NORTON 38A150L6VH aluminum oxide (355.6 × 25.4 × 127 mm)
Dressing tool	CDV and natural diamond single-point conical type dressers
*U_d_*	1 at beginning
Traverse dressing feed *f_d_*	3.45 mm/s
Dressing depth *a_d_*	40 µm
Cooling	Dry-dressing
Number of dressing passes	600 for CVD diamond and 300 for natural diamond
Temperature monitoring	MINIPA MT455 using a type K thermocouple
**State wear analysis**	
DAQ system	NATIONAL NI USB-6221 DAQ device
Transducers	Two diaphragms MURATA 7BB-20-6

**Table 2:** Selected frequency sub-ranges for damage metrics calculation. (RMSD: root mean square deviation; CCDM: the correlation coefficient deviation metric)

Nomenclature	Frequency Sub-Range for RMSD and CCDM Calculation
*@fb#1*	0–20 kHz
*@fb#2*	20–40 kHz
*@fb#3*	40–60 kHz
*@fb#4*	60–80 kHz
*@fb#5*	80–100 kHz
*@fb#6*	100–120 kHz

**Table 3:** Main characteristics of the ANN models in experimental test case #1.

Input Models		Combined Frequency Bands					
		*@fb#1*	0–20 kHz	*@fb#3*	40–60 kHz	*@fb#5*	80–100 kHz
		*@fb#2*	20–40kHz	*@fb#4*	60–80 kHz	*@fb#6*	100–120 kHz
PZT#1 CVD RMSD	*model*#1	1.7%		1.1%		9.8%	
PZT#1 CVD CCDM	*model*#2	14.5%		5.9%		2.6%	
PZT#2 CVD RMSD	*model*#3	17.6%		0.9%		11.9%	
PZT#2 CVD CCDM	*model*#4	2.6%		5.2%		1.9%	
Average error		9.1%		3.2%		6.5%	
Standard deviation		8.1		2.6		5	

**Table 4:** Main characteristics of the best ANN models in experimental test case #1.

Parameters	Specification	
Best input model of the test	PZT#1 CVD RMSD-*model*#1	PZT#2 CVD RMSD-*model*#3
Structure (layers and neurons)	2-10-10-05–3	2-05-15-00-3
Input pattern	[*@fb#3 + @fb#4*]	
Optimal combined frequency ranges	[40–60 kHz + 60–80 kHz]	
Training function	LVM backpropagation	
Max number of epochs	2000	
Number of patterns considered	2000 features for each condition, 10% for validation	
Decision rule of *k*-NN	K=1	

**Table 5:** Main characteristics of the ANN models in experimental test case #2.

Input Models		Combined Frequency Bands					
		*@fb#1*	0–20 kHz	*@fb#3*	40–60 kHz	*@fb#5*	80–100 kHz
		*@fb#2*	20–40kHz	*@fb#4*	60–80 kHz	*@fb#6*	100–120 kHz
PZT#1 natural RMSD	*model*#5	45.2%		1.1%		9.3%	
PZT#1 natural CCDM	*model*#6	1.7%		4.8%		2.4%	
PZT#2 natural RMSD	*model*#7	1.7%		0.6%		8.9%	
PZT#2 natural CCDM	*model*#8	2.8%		5.0%		2.6%	
Average error		12.8%		2.8%		5.8%	
Standard deviation		21.5		2.3		3.8	

**Table 6:** Main characteristics of the best ANN models in experimental test case #2.

Parameters	Specification	
Best Input model of the test	PZT#1 CVD RMSD-*model*#5	PZT#2 CVD RMSD–*model*#6
Structure (layers and neurons)	2-05-05-05–3	2-05-15-00-3
Input pattern	[*@fb#3 + @fb#4*]	
Optimal combined frequency ranges	[40-60 kHz + 60-80 kHz]	
Training function	LVM Backpropagation	
Max number of epochs	2000	
Number of patterns considered	2000 features for each condition, 10% for validation	
Decision rule of *k*-NN	K=1	
